# Review of Rat (*Rattus norvegicus*), Mouse (*Mus musculus*), Guinea pig (*Cavia porcellus*), and Rabbit (*Oryctolagus cuniculus*) Indicators for Welfare Assessment

**DOI:** 10.3390/ani13132167

**Published:** 2023-06-30

**Authors:** Shari Cohen, Cindy Ho

**Affiliations:** 1 Melbourne Veterinary School, Animal Welfare Science Centre, University of Melbourne, Parkville 3010, Australia; dr.cindyho.vet@gmail.com; 2 School of Life and Environmental Sciences, University of Sydney, Camden 2570, Australia

**Keywords:** animal welfare, animal welfare indicators, animal welfare assessment, animal welfare monitoring, rabbit, guinea pig, mice, mouse, rat

## Abstract

**Simple Summary:**

The monitoring and assessment of animals is important for their health and welfare. The appropriate selection of multiple, validated, and feasible welfare assessment indicators is required to effectively identify compromises or improvements in animal welfare. Animal welfare indicators available can be animal based or resource based and collated together to form various assessment tools. The literature contains a wide variety of indicators; however, there is yet to be an ideal constellation of indicators for animal-based welfare assessment in small mammals. A systematic review was performed to identify and outline common animal-based indicators for animal welfare assessment in small mammals, specifically guinea pigs (*Cavia Porcellus*), mice (*Mus musculus*), rabbits (*Oryctolagus cuniculus*), and rats (*Rattus norvegicus*). This review aims to provide the basis for further research into animal welfare indicators for these species and their application to improve animal welfare assessment, management, and strategies.

**Abstract:**

The monitoring and assessment of animals is important for their health and welfare. The appropriate selection of multiple, validated, and feasible welfare assessment indicators is required to effectively identify compromises or improvements to animal welfare. Animal welfare indicators can be animal or resource based. Indicators can be collated to form assessment tools (e.g., grimace scales) or animal welfare assessment models (e.g., 5 Domains) and frameworks (e.g., 5 Freedoms). The literature contains a wide variety of indicators, with both types needed for effective animal welfare assessment; however, there is yet to be an ideal constellation of indicators for animal-based welfare assessment in small mammals such as guinea pigs (*Cavia Porcellus*), mice (*Mus musculus*), rabbits (*Oryctolagus cuniculus*), and rats (*Rattus norvegicus*). A systematic review of grey and peer-reviewed literature was performed to determine the types of animal-based welfare indicators available to identify and assess animal health and welfare in these small mammals maintained across a wide variety of conditions. The available indicators were categorised and scored against a selection of criteria, including potential ease of use and costs. This review and analysis aim to provide the basis for further research into animal welfare indicators for these species. Future applications of this work may include improvements to animal welfare assessments or schemes, guiding better management, and implementing future strategies to enable better animal welfare.

## 1. Introduction

Appropriate animal welfare is foundational to the care and management of animals [1,2]. Animal care and use industries worldwide should be committed to providing high standards of welfare for all animals under their care [3]. There is growing public interest in animal welfare across all areas, including animal care, housing, and use, and increasing interest in evidence-based welfare assessments by veterinary professionals, researchers, animal carers, and stockpersons [4,5]. The standard of animal welfare can currently vary based on the purpose and species of the animal. Each species has its own specific inputs and strategies required for good welfare. During their life, animals may be exposed to a range of positive or negative experiences via human–animal interactions (i.e., handling), husbandry (i.e., diet), management practices (i.e., housing), disease (i.e., preventative care), and other inputs. The acknowledgement of sentience in many species has emphasised the need to ensure and assess good animal welfare through science-based evidence. Although there has been significant growth in animal welfare science, there are still gaps to be explored. One specific gap is in the assessment of animal welfare. There are several methods available for animal welfare assessment, but species-specific indicators are yet to be comprehensively identified, collated, and analysed [3,6]. Efforts have been undertaken in the development and harmonisation of animal welfare indicators for dogs, cats, some farm animals, and zoo animals. Some of the available indicators have been validated and may form part of wider welfare assessments or tools [7,8,9,10,11,12,13,14,15,16,17]. Guinea pigs, mice, rabbits, and rats are commonly part of the pet ownership and laboratory animal industries. The ability to assess welfare more easily, objectively, and consistently in these animals is likely of interest to many animal care staff, pet owners, researchers, veterinarians, and others committed to the well-being of these animals.

The term “animal welfare” is used to describe the affective state of an individual animal, which goes beyond the perception, response, and ability to cope in different circumstances [1,2,18,19,20]. A contemporary and holistic approach to animal welfare is demonstrated in the Five Domains model, where good welfare is defined as an animal that is healthy, comfortable, well nourished, safe, can express behaviours for its physical and mental state, and attains an overall positive welfare experience [21,22,23]. Both positive and negative experiences of an animal are included, with good welfare achieved through the fulfilment of nutritional, physiological/physical, behavioural, and environmental requirements [18]. These experiences are often defined in terms of the affective states of an animal. Affective states are generally described as feelings, emotions, or moods and can include fear, pain, frustration, happiness, and satisfaction [24,25]. These affective states or experiences are explicitly included in the Five Domains model for animal welfare assessment [23,26,27] where animal welfare is considered a continuum of an animal’s affective states or experiences from positive, neutral, to negative [24,25]. Affective states incorporate behavioural, physiological, and cognitive components and are based on two dimensions: level of arousal, which indicates the level or strength of bodily activation (e.g., excited versus relaxed), and valence of the stimulus, which indicates the direction of the stimulus (e.g., positive versus negative) [24,25,28].

Using this approach, the affective state can be used to infer an animal’s welfare state [24,29,30]. For instance, animals are described as having good welfare when they are mainly experiencing positive states, such as pleasure and satisfaction. Conversely, poor welfare can occur when animals mainly experience negative states, such as fear and pain [24,29,30]. Animal welfare can be assessed over the long term and is influenced by a collection of affective states. Alternatively, it can also be viewed as a point in time and monitored with short-term observations [30]. For example, grimace scales can be used as a short-term observation tool to assess the presence and potential severity of pain in animals [31,32,33].

The assessment of animal health and welfare is an important component of veterinary medicine and research. Accurately assessing the welfare of animals is crucial and can be beneficial beyond simply maximising or maintaining a positive state of well-being. Accurate assessment of the welfare of companion animals by veterinarians can potentially increase owner awareness by improving their level of reflection with regards to their pets’ potential issues [34]. Regular welfare assessments can improve the prospect of detecting signs of pain or distress rapidly, consistently, and accurately, so appropriate intervention, such as timely administration of pain relief, is achieved [19]. In research, accurate welfare assessment can mean better detection if an intervention has an impact on animal well-being [35,36]. The routine assessment of animal welfare for research purposes supports the collection of high-quality scientific data [6]. Finally, assessing animal welfare offers an opportunity for continual improvements and a deeper understanding of the requirements of animals to help optimise housing and husbandry practices [19].

Animal welfare is generally assessed through a variety of means. As highlighted by Fraser (2008), the assessment of animal welfare (or the type of welfare assessment model or tools used) can be influenced by different definitions of “animal welfare” and which aspects or concepts of animal welfare are emphasised within the definition [26,37,38]. For the purposes of this paper, this review will use the affective states approach as the primary determinant of animal welfare [25]. Despite there being no direct measures of affective states in animals, affective states can be used to infer animal welfare states. Therefore, welfare measurements rely on drawing inferences from affective states, which are based on assessing animal-based welfare indicators, such as physiological, behavioural, and health indicators [19]. 

Animal welfare assessments should use a combination of resource-based and animal-based indicators [19]. Resource-based welfare indicators are closely associated with animal-based indicators and should be considered when assessing animal welfare, as environmental factors, human–animal interactions, and husbandry all play important roles in the welfare of an animal. However, using only resource-based assessments may not consistently correlate with the affective state of an animal [28]. Consequently, for this review, the evaluation of resource-based indicators has been excluded. Although some publications referenced in this paper may include these indicators, the focus of the review and analysis is on animal-based welfare indicators. To date, there has been no systematic review to appraise animal-based welfare measures, with an emphasis on physiological, behavioural, and physical health indicators for guinea pigs (*Cavia porcellus*), mice (*Mus musculus*), rabbits (*Oryctolagus cuniculus*), and rats (*Rattus norvegicus domestica*). The animal-based welfare indicators found in this review were collated and analysed in regard to their potential practicality of use, such as time, training, cost, and equipment requirements. The analysis explored the breadth, depth, development, and application of animal welfare assessment indicators with the aim of identifying and providing options for their use and direction for future research.

## 2. Materials and Methods

### 2.1. Search Strategy and Eligibility Criteria

**Figure 1:**
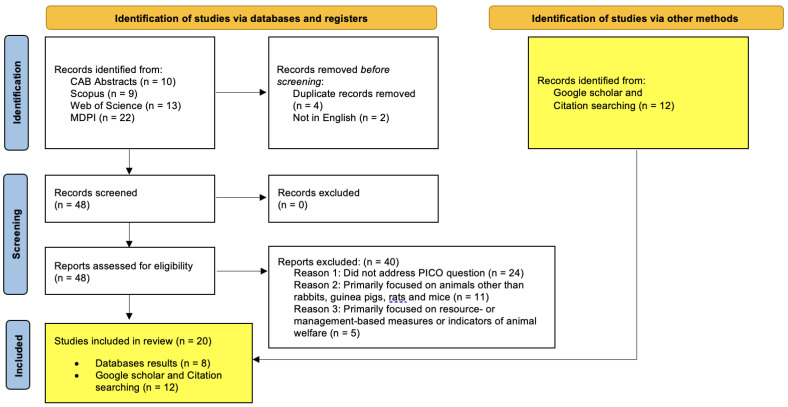
Rabbit focused database searches—PRISMA flow diagram for articles sourced from CAB Abstracts, Scopus, Web of Science and MDPI databases using the following terms: TOPIC: (rabbit) AND/OR TOPIC: (welfare) AND/OR TOPIC: (welfare indicator) AND/OR TOPIC: (welfare assessment).

**Figure 2:**
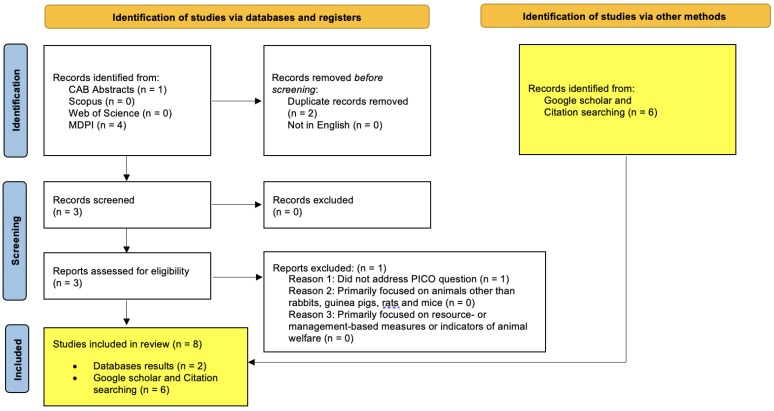
Guinea pig focused database searches—PRISMA flow diagram for articles sourced from CAB Abstracts, Scopus, Web of Science and MDPI databases using the following terms: TOPIC: (guinea pig) AND/OR TOPIC: (welfare) AND/OR TOPIC: (welfare indicator) AND/OR TOPIC: (welfare assessment).

**Figure 3:**
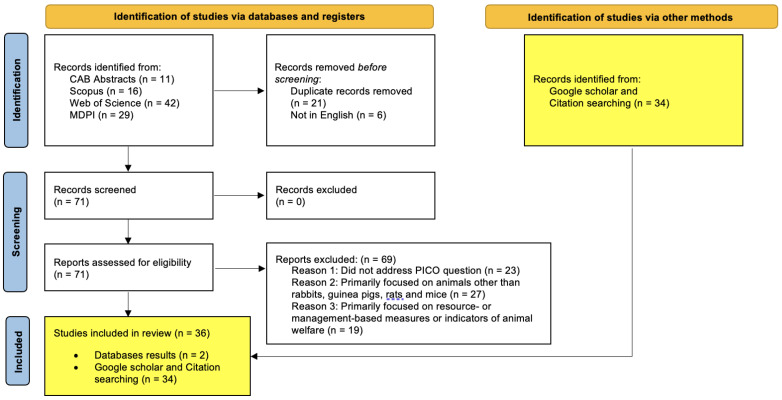
Rat focused database searches—PRISMA flow diagram for articles sourced from CAB Abstracts, Scopus, Web of Science and MDPI databases using the following terms: TOPIC: (rat) AND/OR TOPIC: (welfare) AND/OR TOPIC: (welfare indicator) AND/OR TOPIC: (welfare assessment).

**Figure 4:**
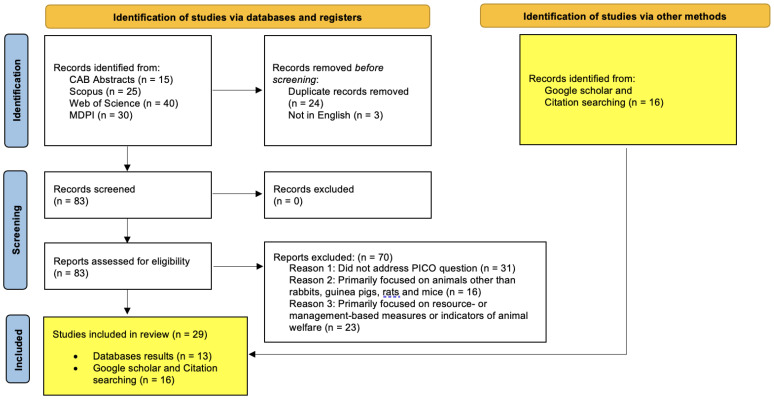
Mouse focused database searches—PRISMA flow diagram for articles sourced from CAB Abstracts, Scopus, Web of Science and MDPI databases using the following terms: TOPIC: (mice or mouse) AND/OR TOPIC: (welfare) AND/OR TOPIC: (welfare indicator) AND/OR TOPIC: (welfare assessment).

The article analyses the current literature with regards to animal-based welfare measures in accordance with PRISMA guidelines [39]. A systematic search was undertaken using four independent databases on each of the four animals of interest (rabbits, guinea pigs, rats, and mice). The four databases used in these searches were: CAB Abstracts, Scopus, Web of Science, and MDPI (Multidisciplinary Digital Publishing Institute). A systematic search was performed on these databases using combinations of keywords: “welfare” and/or “welfare indicator” and/or “welfare assessment”, and/or “rabbit”, and/or “guinea pigs”, and/or “rats”, and/or “mice”, and/or “mouse”. Databases were searched as per the PRISMA guidelines using the eligibility criteria listed in Table 1 and the results described in Figure 1, Figure 2, Figure 3 and Figure 4. The PRISMA diagrams in Figure 1, Figure 2, Figure 3 and Figure 4 have been modified to account for the presence of grey literature. The search terms and results for each database search are presented in Figure 1, Figure 2, Figure 3 and Figure 4. The title and abstract of each citation were inspected to identify suitable articles based on the inclusion and exclusion criteria listed in Table 1. Hand-picked key texts or articles used by the authors in veterinary medicine and teaching were also selected alongside those found in Google Scholar. All database and hand-picked searches were conducted between April and August 2022, and there were no restrictions on the publication date; however, if there were two or more versions of any publication, the more recent publication was selected.

### 2.2. Analysis of Indicators from the Literature

Publications meeting all eligibility criteria were reviewed. All animal-based welfare indicators found were analysed and placed into three separate tables labelled Table 2 with sections for rabbits (A), guinea pigs (B), as well as rats and mice (C). Due to the large proportion of shared or similar animal-based welfare indicators, rat and mouse welfare indicators were combined into a single table. Indicators were grouped into three categories: physiological, behavioural, and physical health indicators. Each category was subcategorised into its respective body systems, body parts, organs, or descriptors. An additional five parameters were included to denote the potential welfare state, ease, and practicality of the indicators. Animal welfare indicators were linked with a possible associated positive, neutral, or negative affective state (i.e., welfare state). In addition to affective state, four other parameters described the potential practicality and ease of use of the indicators in terms of “easy training”, “specialized equipment” required, approximate equipment and/or associated “costs”, and approximate “time to assess” the indicator. Training was deemed easy or not easy as a “yes or no” if it was thought it could be taught to a layperson or student within a 15 min consultation. Cost was categorised as “yes” or “no” and was deemed “high” if it cost more than AUD 100.00 to purchase the equipment or required the test to be performed in a specialised setting such as a laboratory or veterinary clinic. Special equipment was denoted “yes” or “no” if any equipment was required to undertake the assessment. Time was categorised as “yes” or “no” if it was likely to require more than five minutes to perform the assessment. Note that the allocated affective states as well as the ease and practicality criteria of each indicator in Table 2 are semi-subjective.

## 3. Results

### 3.1. Animal Welfare Based Indicators

This study identified a total of 190 animal-based welfare indicators across all four species. Of these 190 indicators, a total of 75 welfare indicators were found to be exhibited by rabbits. For guinea pigs, a total of 49 welfare indicators were identified. For rats and mice, a total of 66 welfare indicators were identified. Most of the indicators discovered were behavioural indicators, totalling 99 indicators. Physical health indicators totalled 52, and the fewest indicators were physiological, with a total of 39 indicators. Table 3 provides a further breakdown of the number of indicators identified for each animal based on the three categories: physiological, physical health, and behavioural.

From these 190 indicators, there were a total of 9 physiological, 8 physical health, and 10 behavioural shared welfare indicators across all 4 species. Shared indicators are listed in Table 4. 

Based on the literature screened, studies infrequently focused on either the direct assessment of welfare or the validation of animal-based welfare indicators. Studies frequently aimed to evaluate non-animal welfare-based indicators such as management, environmental, or other resource-based factors as well as monitoring behaviour. Some studies focused on the physiological response to various stimuli for non-animal welfare purposes, such as pain studies and stress in biomedical research. Nonetheless, these studies were included and evaluated, as the findings can be utilised to assess aspects of animal welfare and strengthen the validity of certain indicators. 

### 3.2. Animal Welfare Indicator Scoring Systems

While reviewing relevant publications, two commonly used scoring systems were found to be used for recording welfare indicators. Systems were either binary or numerically recorded, with their relative strengths and weaknesses outlined in Table 5.

## 4. Discussion

### 4.1. General Discussion

There is a need to study and identify best practices and new welfare assessments via the use of validated welfare indicators. The overall societal interest in animal welfare issues has increased, with a likely reciprocal desire among veterinarians, researchers, pet owners, and animal care staff to deliver improvements in animal welfare. Guinea pigs, mice, rabbits, and rats may be exposed to a range of stressful stimuli in their lifetime, such as transport, disease, poor husbandry, management, and/or experimental conditions, which can all disrupt their state of welfare and may result in an overall negative cumulative effect. While avoiding all stress may not be feasible or necessary to maintain an overall positive state of welfare, the ability to detect pain, stress, or distress enables appropriate interventions to alleviate or mitigate negative states of welfare. Most importantly, when determining the welfare state of animals, a carefully considered and multi-factorial approach is required. 

This study has identified a range of animal-based welfare indicators, demonstrated in Table 1, that can or are currently being utilised as components of welfare assessment tools for guinea pigs, mice, rabbits, and rats. The systematic review and analysis of the literature found a selection of common welfare indicators shared amongst these species. In addition, many of these indicators are assessments of animal health, which is an important pillar of animal welfare; changes in these indicators may not correspond to changes in welfare. Thresholds for abnormalities and baselines of normality should also be considered when determining if changes in animal health indicators are potentially significant. The categories selected denote three main themes of welfare indicators, each with its own advantages and disadvantages. These indicators are of potential interest to those involved in the care and use of animals, including researchers, veterinarians, students, pet owners, and animal care staff. The tables highlight the breadth and depth of animal welfare assessment indicators and key considerations in their usage. This information may provide foundational support in future animal welfare assessment schemes for educators and animal inspectors in selecting appropriate species-specific and more generic animal-based indicators in these different contexts. The review and analysis of these indicators across these species were also able to highlight knowledge deficiencies across the different categories, types of indicators, and potential limitations that may provide direction for future research and development. 

Animal welfare is widely studied across many species. Animal carers, animal use industries, and governments are moving away from the prevention of cruelty to the provision of positive animal welfare experiences. Critical to achieving this shift is the ability to identify positive and negative welfare states through appropriate monitoring and welfare indicators. However, there is currently no consensus regarding the animal-based welfare indicators to be assessed when evaluating the welfare of small mammals such as guinea pigs, mice, rabbits, and rats. Systematic review of the current literature demonstrated a large discrepancy in the quality and quantity of information provided by scientific articles and other literature. While a large portion of welfare indicators were found in the grey literature, such as in veterinary textbooks, on veterinary websites, pet websites, animal welfare society websites, and similar guidelines, only a small portion of welfare indicators were addressed in scientific papers. This suggests that welfare indicators may be selected based on anecdotal evidence and/or expert opinion. It is unknown if there may be issues or limitations with non-evidence-based or non-animal-specific indicators being used to assess the well-being of other animals. 

Animal welfare assessment schemes can be derived from existing animal welfare frameworks such as the 5 Freedoms and 5 Domains and can provide a high-level approach to animal welfare assessment [22,23]. In guinea pigs, mice, rabbits, and rats, there are few publications available for validated, formal animal welfare assessments and no known work that outlines current utility. For rabbits, a recent study by Botelho and colleagues (2020) aimed to assess the welfare of commercially reared rabbits. These researchers developed a set of animal welfare assessment protocols based on the Welfare Quality Approach by using existing animal welfare indicators obtained from other studies [46]. In mice, a study by Campos-Luna and colleagues (2019) aimed to determine the most valid, reliable, and practicable welfare indicators for laboratory mice. The researchers in this study applied the Delphi consultation technique as a method of finding consensus amongst experts for mice [72]. There were no other similar studies involving rats and guinea pigs. This is of concern, given that guinea pigs are used as a food source in some countries, and both rats and guinea pigs are used in research and as companion animals. Although many mouse indicators are used to assess welfare in rats given their similarities in physiology, physical features, and behaviours, the tables demonstrated the important nuanced differences between the species. In the case of guinea pigs, there was an abundance of information found on pet websites but few scientific sources detailing guinea pig welfare assessment parameters, highlighting key gaps in scientific knowledge.

Some research companies and international animal research councils have developed publicly available welfare assessment procedures [19,67,68,131]. For example, Wageningen UR Livestock Research (2011) published a guideline for the assessment of commercially housed rabbits with potential animal- and resource-based measures based on the Welfare Quality Approach [131]. Other guidelines include the Animal Welfare Guidelines for Rats and Mice published by the Canadian Council on Animal Care (CCAC), which suggest a variety of welfare assessment tools for laboratory rats and mice [19,67,68]. These guidelines were useful in providing subsets of welfare indicators for analysis in this review. However, caution should be used when applying and assessing these indicators in other contexts, as many have been extracted from studies conducted on laboratory and commercial animals for non-animal welfare purposes, with many yet to be validated. For guinea pigs, the literature on welfare assessments is very sparse and still limited. The disparity in animal welfare knowledge and literature between the species, particularly for guinea pigs, is further supported in Table 2, which demonstrates guinea pigs possessed the fewest number of animal-based welfare indicators. Overall, there is a dearth across all species for life stage-specific indicators such as those for neonates, during pregnancy, or in geriatrics.

Animal welfare and assessment is multi-dimensional, and various welfare assessment frameworks have generated multiple tools; however, these tools are often developed for specific species or contexts [132]. As such, there is no universal approach for assessing animal welfare [132]. A total of 27 shared animal-based welfare indicators for different species were found. These similarities are listed in Table 3. There were numerous indicators that were also potentially specific to each species. For example, tooth purring or tooth chattering, which can be heard in content or sleeping rabbits but should not be confused with loud tooth grinding (bruxism), can also be heard in rabbits suffering from severe pain or discomfort [40,51,52]. Other indicators specific to rabbits and rodents (e.g., rats and mice) included burrowing/digging [52,74,86] and pain-related indicators such as facial grimacing [31,32,33,53] and altered posture [40,69,84]. Future studies developing animal welfare assessment protocols for small mammals could address the lack of harmonisation of these indicators to create a standardised list of key general welfare and species-specific assessment parameters via the list of indicators identified by this study.

### 4.2. Welfare Indicator Categories

Animal-based and resource-based assessments are frequently used simultaneously in many animal welfare assessment programs [19,46]. This holistic approach facilitates comprehensive yet feasible assessments; however, animal-based welfare indicators must be a prime focus of any welfare assessment program [19]. Animal-based welfare indicators are important as they directly assess the response (outcome) of an animal to its environment through physiological, physical, and behavioural measures [28,132,133]. Conversely, resource-based indicators evaluate indirect elements such as the animal’s environment and quality of husbandry. Both types of indicators play an important role in optimising and benchmarking acceptable practices for animal environments, husbandry, and overall animal welfare [133]. 

For the purposes of this review, animal-based welfare indicators were divided into three broad categories: Physiological indicators (Section 4.2.1), physical appearance or observable health indicators (Section 4.2.2), and behavioural indicators (Section 4.2.3). Together, these categories provide insight into the welfare of animals in a practical and sometimes scientific context. However, each category possesses advantages and disadvantages concerning its practical application.

#### 4.2.1. Physiological Indicators

Physiological indicators can be used to detect potential stress responses in animals and can be assessed directly via vital signs or by analysing bodily fluids. These vital signs, such as heart rate (HR), respiratory rate (RR), blood pressure (BP), and body temperature, can represent a critical baseline of vital indicators of animal health and welfare [134]. The activation of the hypothalamic–pituitary–adrenal (HPA) axis in a stressful event creates a cascade of reactions, including changes in the secretion of glucocorticoid hormones from the adrenal glands [135]. The secretion of hormones and their metabolites, such as cortisol and corticosterone (in rodents), can be measured in different types of bodily fluids [33]. For example, paired or multiple blood plasma, serum, and saliva samples can be used to identify states of acute stress, while fur, urine, and faeces can be used to determine chronic stress [62,136,137,138,139]. Although these methods may be useful to indicate stress and potential welfare compromise, ongoing or repeated monitoring of these indicators is not always feasible, financially viable, or timely, which may limit their use. Physiological parameters must also be interpreted in context with the type of stress (distress vs. eustress). Nonetheless, deviations from baseline values should be cause for further review and assessment to ensure animals are not experiencing unnecessary negative affective states. 

Laboratory physiological indicators can provide an abundance of information about an animal’s welfare. However, their applicability in practical and scientific contexts may be limited due to several reasons. The assessment of physiological indicators is often more invasive and disruptive compared to the assessment of other types of indicators. Physiological indicators such as serum and plasma cortisol levels are considered invasive as they require venepuncture for the collection of blood. Depending on the purpose of the study (i.e., determination of acute versus chronic stress), appropriate alternatives and substitutes to blood sampling can include fur, saliva, urine, or faecal sampling [42,43,48,49,62,69,72,78,79,139]. However, the collection of saliva and fur may be invasive based on the circumstances under which the samples are obtained (i.e., directly from the animal). The collection of urine and faeces from the enclosure may also be disruptive to the animal for similar reasons. In addition to physical contact, the collection of animal samples often requires animal handling and restraint. Sampling and animal handling procedures can act as stressors and affect cortisol concentrations, which can cause confounding issues when assessing acute stress [140,141]. On the other hand, some animals may be accustomed to handling and sampling procedures, and therefore, care must be taken when comparing and interpreting these results. Significant investment is required when analysing laboratory parameters, as this process can be costly, time consuming, and require specialised equipment. 

Vital signs are considered a critical baseline set of indicators in the assessment of animal health and welfare. Persistent elevations in vital signs, including body temperature, BP, HR, RR, respiratory rhythm, effort, and sounds, can be early indicators of heat stress, disease, or other physiological complications [142]. These indicators can also be associated with the mental states of anxiety, fear, pain, discomfort, and distress [142]. The assessment of RR is highly recommended as it is easy-to-assess, non-invasive, and chest movements can be observed from a distance. On the contrary, the assessment of HR, BP, and body temperature requires handling, physical restraint, and contact with the animal. As guinea pigs, rabbits, and rodents are extremely sensitive to physical contact, restraint, and handling, transient changes in vital signs may be a confounder. Some technologies, such as the use of telemetry sensor implants in rodents, have been developed to try and avoid these issues, but they may be more aversive, thus negating their use [142]. Newer unobtrusive and contactless modalities for monitoring and assessing physiological parameters are being explored, which include the use of infrared thermography and motion tracking technologies [142,143,144,145]. The benefits of using these technologies are that monitoring is remote and passive, causing little to no disturbance to the animal and avoiding manual assessment of vital signs [142]. However, as these technologies are expensive, their use may not be limited at this time.

A primary advantage of physiological indices of assessment is the ability to measure and quantify animal responses [146]. The disadvantages are the existence of unlimited variability in physiological responses, contextualisation (e.g., heart rate increase due to excitement from feeding versus fear), and potential invasiveness [146]. Physiological responses can be influenced by factors such as species differences, age, sex, body condition, reproductive status, the animal’s history and past experiences, the animal’s mental state, geographic location, current energetic status, and the circumstances under which samples are collected [147]. In addition, examining and interpreting physiological indicators requires training, expertise, knowledge, and skills. This can potentially make comparisons and interpretations difficult even among experts in an academic context, which can prevent the common practical use of many of these indicators. Future research should focus on the further characterisation of non-invasive sampling techniques for practical use in the field and in real time. Apart from respiratory rate, non-invasive and real-time sampling methods are not commonly available for many of these techniques and may not yet be practical for routine welfare assessments. 

#### 4.2.2. Physical Health Indicators

Physical appearance and physical health measures are indicators of welfare that can be obtained through cage-side or pen-side visual observation and/or physical examination of an animal. Assessment of physical health and appearance may involve evaluating animal body weight, body condition score, and observing for signs of abnormalities such as physical injuries and/or discharges. It can also include observing physical signs of pain via alterations in posture. For some animals, pain can be scored via validated pain scoring systems such as facial grimace scales. Although assessing physical health and appearance is generally non-invasive, some indicators, such as body weight and body condition score, involve handling and manipulation of the animal. The weighing procedure also requires the use of a scale, which, for small animals, may be quite simple to use or access. Overall, the assessment of physical health using indicators such as body weight and body condition scores can be relatively quick, easy, and inexpensive and does not usually involve complex training.

A key disadvantage in evaluating physical appearance and observable health indicators of welfare is that many small mammals, including guinea pigs, mice, rabbits, and rats, are prey animals. Prey animals may be more likely to hide or minimally express clinical signs of disease, injury, weakness, and pain to elude predators and/or humans [148,149]. The absence of abnormal physical health indicators does not necessarily mean an animal is healthy, pain-free, and in a positive state of welfare. Issues may also arise when attempting to notice subtle deviations in health deterioration in group-housed laboratory animals, normally social animals that are individually housed, or when social hierarchies are disturbed. Additionally, while resource-based indicators of group housing, more space, and places to hide can provide potential welfare benefits, these elements may also limit direct visual inspection of some animals, especially in shy, stressed, or injured individuals [148]. A balanced (and creative) approach between potentially positive resource-based requirements and the need to use animal-based indicators to assess welfare should always be applied. 

Unlike the assessment of physiological parameters, observing physical appearance is a non-invasive and effective method of contributing to the assessment of animal welfare. Several validated tools for visually detecting pain have been developed, including the Composite Pain Scores and Facial Grimace Scores [31,32,33,53]. Facial Grimace Scales are a specialised example of a physically observable assessment tool that can be used as a valid and reliable method for determining the presence of pain in rabbits, rodents, and other animals [31,35,40,150,151,152,153]. By observing simple changes in facial expression, valuable information can be provided on animal affective experiences/states [31,32,33,53]. However, like many other observable indicators, it must be interpreted in context and with other indicators to confirm negative states (i.e., pain) [36,153]. For example, rats in severe pain can demonstrate a pain face in conjunction with a hunched posture and tucked abdomen [40,84], but they can also express these signs during recovery from anaesthesia, aggression, and sleep [32,33,154,155]. As suggested previously, the application of various imaging, scanning, or video technologies may be beneficial when assessing the physical state of an animal to determine its mental state [32,53,156,157]. At times, the use of advanced technologies may not be feasible. However, human observation of physical health, paired with regular examinations (e.g., BCS, weights), can be critical in detecting early concerns about animal welfare. The provision of visual aids (e.g., video, photos) and appropriate staff training is also likely to enhance the application of these possible welfare-improving techniques. The appropriate selection of physical health indicators and the best way to train humans in the use of these indicators are areas warranting additional work to further advance animal welfare and assessment. 

#### 4.2.3. Behavioural Indicators

Behavioural indicators of welfare were found to be the most common and well-studied of the three welfare indicator categories. This is demonstrated by the large proportion of behaviour-based welfare indicators in Table 1 compared to physiological and physical health indicators. This is perhaps because they are the easiest to assess in both practical and experimental contexts. As behavioural indicators of welfare are normally based on observations, they might be the most minimally invasive and least disruptive of the three welfare indicator categories. Behavioural indicators were subcategorised into natural and abnormal behaviours. There is a collection of several natural behaviours that may be used to infer a positive affective state [158]. Natural exploratory behaviours such as foraging, sniffing, burrowing, play, object manipulation/approach, resting, laying down, and social interactions such as mutual grooming. Abnormal behaviours encompass a range of abnormal repetitive or stereotypic behaviours, such as excessive scratching or rubbing, as well as behaviours related to states of disease or pain, such as reluctance to move, lack of responsiveness, and failure to groom or overgrooming. However, behavioural abnormalities can be complex. For example, an animal demonstrating abnormal behaviour may have used this behaviour as a coping mechanism in a barren environment but may continue this “learned” behaviour even after being moved into an appropriate enriched environment. Therefore, caution may be warranted, along with a good understanding of prior negative experiences and context, when assessing affective states.

Challenges to incorporating behavioural indicators into welfare assessments include, but are not limited to, general interpretation and difficulties in assessing animals in some scenarios. A holistic approach is important for animal welfare assessments, as utilising a single welfare indicator separately may be inaccurate [140]. The use of a few incorrectly chosen indicators can be misleading, and welfare compromise can exist in the absence of behavioural, physiological, or physical changes [146]. Additionally, some behaviours, such as mating, may be life-stage or time-sensitive specific and should (or can) only be assessed at specific times [158]. 

Another consideration is that interactions between social, exploratory, and play behaviours may be difficult to assess in animals captively managed in complex environments or intentionally isolated for short durations (e.g., transport or veterinary care). It may take time and familiarity to distinguish normal vs. abnormal use of hiding holes or shelters due to pain or stress in animals [150]. Separation of social animals from their conspecifics may also result in temporary behavioural changes. Several publications have found that social isolation can induce (locomotor) hyperactivity in adult rats; however, when returning previously isolated rats to their group, this behaviour was ameliorated [104,105]. Either set of changes may be interpreted as positive or negative behaviour, pending the social hierarchies and/or nuances of the animal and the environment. Consequently, careful interpretation and context are needed when assessing the welfare of individual animals surrounded by a group of animals of the same species, and vice versa. An additional characteristic that may be useful to explore and apply further is determining when animal welfare indicators should be used and if they are applicable to individual and/or population-based assessments. 

### 4.3. Animal Welfare Scoring Systems

When working with animal welfare indicators, the scoring system has the potential to affect scores and animal welfare outcomes. Indicators can be scored and recorded via a binary or numerical system. In the case of the binary system, a simple “yes” or “no” is recorded to denote the presence or absence of an indicator [158]. This type of scoring system is simple yet effective when more detailed information or scaled data is not required. However, this type of scoring system may not be appropriate when a gradient of scoring is required or if the indicator/question is not suitable for binary scoring. If this occurs, it may be difficult to determine whether the score is valid and/or result in the loss of valuable information.

In contrast, a numerical system offers the possibility of scoring severity and/or intensity [158]. Numerical scores can offer a better opportunity to track trends and provide a greater depth of information. Simple versions of the numerical scores found in Table 2 included weight and BCS. It is tempting to collectively sum numerical scores from various animal welfare indicators to give a total score to denote “good” or “bad” welfare or offer a strict numerical delineation to determine when intervention or further action is required. However, the summation of scores may not always be accurate or justified and may sometimes prove inadvertently detrimental to animal welfare by reducing sensitivity and/or specificity. Additionally, not all indicators are necessarily equally important to animal welfare, and a weighted score may be more appropriate. 

When developing or using a scoring system for animal welfare assessment, it is essential to have appropriate language, minimal overlap between outcomes, training of high-quality assessors, and consistency. Accurate, consistent scoring by observers is potentially more likely to occur if assessors are appropriately trained and possess appropriate experience and knowledge of the species being assessed. Assessor attributes, such as teamwork, empathy, and communication, can be as important as the scoring system [158]. The provision of evaluation guidelines with sufficient instructions and pictures can also assist in greater inter-observer reliability [44,104,105]. Consistency can permit comparisons, trend analysis, and benefit animal research and welfare [158]. Scores obtained from either system (i.e., binary or numerical) require careful interpretation and awareness of any limitations. Depending on the indicators and context, either system may be appropriate [94]. A good understanding of both systems and associated factors is important when selecting animal welfare indicators for overall animal welfare assessment and in more formal animal welfare assessment schemes. 

### 4.4. Limitations

The aim of the review was to collate as many known indicators as possible with the understanding that there might be limitations in demonstrating their validity but that they could offer potential areas for future exploration. Limitations in the study include the potential search methodology and the associated number of relevant publications selected and reviewed. Not all publications concerning the objective of the current work may have been identified by the systematic literature search. For example, some publications may not have been found in the databases searched or if indicators or assessment tools used were not stated in the title or abstract (e.g., biomedical studies). Furthermore, due to the vast array of synonyms for the search terms used and the subsequent large number of irrelevant results, keywords and synonyms were sparingly used. Although this technique offered the best return on relevant search results, it may have limited the detection of publications in which the term “welfare” and its synonyms were not included in the title or abstract, thus highlighting the difficulties and nuances of keywords in systematic searches. In addition, the automation tools (e.g., exclusion tools) incorporated into the search databases used to eliminate irrelevant/ineligible papers may also limit the detection of relevant publications. Finally, given the scarcity of available peer-reviewed published papers pertaining to valid, reliable, and feasible animal-based welfare assessment protocols, the tables relied on a higher than preferred portion of grey literature. The combination of utilising primary and secondary sources from grey literature and obtaining information from these global citation databases has proven to be a suitable starting point and approach for the purposes of this review paper.

### 4.5. Future Directions

Key gaps in the literature reviewed demonstrated a lower number of studies and indicators available for guinea pigs and rats, which indicates a potential risk to animal welfare due to a potential lack of validated knowledge. Across the grey literature, mouse indicators are commonly used for rats, but it is unknown if this is accurate and valid for all available indicators as most peer-reviewed publications are centred around mice. Additionally, determining the ease of use of these indicators across a variety of animal care settings (laboratory versus pet) may also be useful. However, there appears to be a baseline of shared indicators that could be useful across all species and other potential species of animals. Future research should potentially be directed towards a deeper understanding of these animals, as this would support a better interpretation of experimental results in animals for both welfare and non-welfare purposes. 

More knowledge about animal welfare, indicators, and the utilisation of collectives of indicators is likely to enhance animal welfare. Moving forward, using the list of welfare indicators identified in this study, future studies should aim to determine the ideal set of welfare indicators for these animals. As the Delphi consultation technique has been shown to be a valuable tool in selecting the ideal set of mouse welfare indicators, applying the same technique to rabbit, guinea pig, and rat welfare indicators may be worth considering. Determining ideal constellations of key general and species-specific indicators could help support better training, education, and standardisation of animal welfare requirements across animal care and use industries and promote better animal welfare for all species.

## 5. Conclusions

In conclusion, animal welfare is an area of increasing interest for animal care and use staff, industry, the research community, government bodies, and the public. It is important for those working with animals to continually enhance animal welfare. It is essential to use evidence-based assessment tools, including animal-based indicators, to evaluate animal welfare. A large number (190) of potential animal-based welfare indicators for guinea pigs, mice, rabbits, and rats and mice were identified. The literature was reviewed and categorised to outline potential animal welfare indicators based on their physiological, physical health, and behavioural elements. This research may be useful in the future when developing assessment protocols. Additional analysis of the possible ease and practicality of their use was performed, reviewing costs, equipment, time, and training requirements. With the help of experts, a more harmonised basic animal welfare assessment system may be developed to better evaluate animal welfare in day-to-day practice as well as in experimental settings. The development of a welfare assessment protocol or welfare scheme from this work should be species specific, well considered (including context), trialled, validated, and recognise negative, neutral, and positive states of welfare to facilitate improvements in animal welfare outcomes. 

## Figures and Tables

**Table 1:** List of inclusion and exclusion criteria for database searches.

Inclusion Criteria	Exclusion Criteria
Publications were considered if they were: - Published in English or translated into English - Primarily focused on rabbits, guinea pigs, rats, or mice - Primarily focused on assessing welfare either directly or indirectly, including: ◦ Animal-based measures or indicators of animal welfare (physiological, behavioural, physical health) ◦ Animal welfare indicators that have been validated - Studies key to the development of methods or tools for assessing welfare in rabbits, guinea pigs, rats, and mice	Publications were not considered if they were: Duplicates Not published in English Did not address PICO question Studies that did not use animal-based welfare indicators as their primary measure of welfare Primarily focused on resource- or management-based measures or indicators of animal welfare

**Table 2:** Animal-based welfare indicators grouped into physiological, behavioural and physical health and practical considerations..

**(A) Rabbit Animal-Based Welfare Indicators Grouped into Physiological, Behavioural and Physical Health and Practical Considerations.**							
**Body-System, Body Part,** **Organ, or Descriptor of** **Indicator**	Animal-Based Welfare Indicators	Affective State	Easy to Train	High Costs (>AUD100)	Special Equipment	Time < 5 min	Publications
**CATEGORY 1: PHYSIOLOGICAL**							
**Cardiovascular system**	Persistent increase or decrease or abnormality in heart rate	Negative	Yes	Yes—stethoscope	Yes—stethoscope	Yes	[40,41,42]
	Heart murmur or arrhythmia	Negative, Neutral	Yes	Yes—stethoscope or electrocardiogram	Yes—stethoscope or electrocardiogram	Yes	[42]
	Poor/weak quality or asynchronous pulses	Negative	Yes	Yes—stethoscope	Yes—stethoscope	Yes	[42]
	Increased blood pressure	Negative	No	Yes—Doppler or oscillometric device	Yes—Doppler or oscillometric device	Yes	[40,42]
	Prolonged capillary refill time	Negative	Yes	No	No	Yes	[42]
**Respiratory system**	Persistent decrease or increase in respiratory rate and effort (laboured breathing/respiratory distress/dyspnoea/apnoea/abdominal breathing)	Negative	Yes	No	No	Yes	[40,41,42,43,44,45]
	Persistent presence of respiratory sounds (including coughing, sneezing, sniffling, wheezes, and crackles)	Negative	Yes	Yes—stethoscope	Yes—stethoscope	Yes	[42,43,45,46]
**Alimentary, gastrointestinal, digestive system**	Reduced gut sounds	Negative	Yes	Yes—stethoscope	Yes—stethoscope	Yes	[42]
	Tender or painful abdomen	Negative	Yes	No	No	Yes	[42]
	Distended abdomen	Negative	Yes	No	No	Yes	[42,45]
	Presence of diarrhoea or soft faeces with an unpleasant smell	Negative	Yes	No	No	Yes	[40,42,43,44,47]
	Changes in faecal output and appearance (including colour, consistency, frequency)	Negative	Yes	No	Yes—measuring utensils	Yes	[40,42,43,44,47]
**Urogenital system**	Changes in urine output and appearance (incl. colour, consistency, frequency)	Negative	Yes	No	Yes—measuring utensils	Yes	[42,44,45]
**Musculoskeletal,** **nervous and vestibular system**	Abnormalities in gait Gait score Abnormalities include lack of balance, stumbling, stiff-legged gait, reduced range of motion or reluctance to move	Negative	No Yes	No	No	Yes	[42,46]
**Nervous system,** **Adrenal glands**	Persistent increase or decrease in glucocorticoid levels and metabolites in comparison to normal.	Negative, Neutral	No	Yes—corticosterone radioimmunoassay, etc.	Yes—corticosterone radioimmunoassay, etc.	No	[41,42,43,48,49]
**Body temperature**	Abnormal changes in body or ear temperature	Negative	Yes	No	Yes—thermometer	Yes	[40,41,42,44,45,46]
**Hydration status**	Dehydration—presence of skin tenting, sunken and dull eyes	Negative, Neutral	Yes	No	No	Yes	[42]
**CATEGORY 2: PHYSICAL HEALTH**							
**Body weight**	Excess weight gain or loss	Negative, Neutral	Yes	No	Yes—scales	Yes	[40,42,43,45]
**Body condition**	Body condition score	Negative, Neutral	Yes	No	No	Yes	[42,43,46,50]
	Body symmetry	Neutral, Negative	Yes	No	No	Yes	[42]
**Integument system**	Unkempt, dirty, matted coat, or urine/faecal stained fur including medial forelimbs	Negative	Yes	No	No	Yes	[40,42,43,45,46]
	Piloerection	Negative, Neutral	Yes	No	No	Yes	[42,51,52]
	Dandruff	Negative, Neutral	Yes	No	No	Yes	[42]
	Damage to fur or skin Includes discolouration, lesions, sores, scabs, scales, ulcerations, crusting or areas of hair loss	Negative	Yes	No	No	Yes	[42,45,46]
	Lumps under chin or mandible	Negative	Yes	No	No	Yes	[42]
	Lumps in or under skin	Negative, Neutral	Yes	No	No	Yes	[42,45,46]
**Eyes, nose, urogenital,** **mammary glands**	Presence of discharge from the eyes, nose, mammary glands, urinary or genital organs	Negative	Yes	No	No	Yes	[42,44,46]
**Musculoskeletal, nervous and** **vestibular system**	Sudden head tilt or loss of balance	Negative	Yes	No	No	Yes	[42,44]
	Facial asymmetry	Negative, Neutral	Yes	No	No	Yes	[42]
	Facial paralysis	Negative	Yes	No	No	Yes	[42]
	Swollen joints	Negative	Yes	No	No	Yes	[42,44]
**Posture**	Hunched back	Negative	Yes	No	No	Yes	[40]
	Tucked up appearance (including tucked abdomen)	Negative	Yes	No	No	Yes	[40,42,45,52]
	Pressing abdomen to the floor	Negative	Yes	No	No	Yes	[40,42,51,52]
**Oral cavity and dentition**	Dental malocclusion, overgrowth, malformation, discolouration and loose, fracture or absent teeth	Negative	No	Yes—otoscope or oral speculum	Yes—otoscope or oral speculum	Yes	[42]
	Bleeding or evidence of trauma to the gingiva	Negative	Yes	Yes—otoscope or oral speculum	Yes—otoscope or oral speculum	Yes	[42]
	Drooling (ptyalism) or wet chin	Negative	Yes	No	No	Yes	[42,45]
	Absences or restricted lateral jaw movement	Negative	Yes	No	No	Yes	[42]
**Eyes**	Wide/partially closed, sunken, or dull eyes	Negative	Yes	No	No	Yes	[40,42]
	Redness, discolouration, masses or swelling of the periocular areas, eyelids, conjunctiva, or sclera	Negative	Yes	No	No	Yes	[42]
	Protrusion of third eyelid	Negative	Yes	No	No	Yes	[42]
**Ears**	Presence of excessive or abnormal coloured wax, ulcerations, scabbing, scaling, discolouration, or swellings	Negative	Yes	Yes—otoscope	Yes—otoscope	Yes	[42]
**Pain-related signs**	Facial Grimacing (Grimace scale) **Grimace scale for Rabbits—Indicators:** Orbital tightening Cheek flattening Nostril shape (“V-shaped” nares) Ear shape and position (ears drawn back) Whisker shape and position	Negative	Yes	No	No	Yes	[31,40,42,44,52,53,54]
	Increased composite pain score	Negative	No	No	No	Yes	[55]
**CATEGORY 3: BEHAVIOUR**							
**Natural behaviours**	Binkying or frolicking (jumping rapidly whilst shaking head and flinging hindlimbs to the side)	Positive	Yes	No	No	Yes	[52,54]
	Grooming (self-grooming, allo-grooming, mutual grooming)	Positive	Yes	No	No	Yes	[40,52,54,56]
	Nocturnal/crepuscular behaviour	Positive	Yes	No	No	Yes	[52,54,57]
	Nesting (for breeding does)	Positive	Yes	No	No	Yes	[52,54]
	Regular eating with occasional drinking	Positive	Yes	No	No	Yes	[52,54]
	Coprophagia	Positive	Yes	No	No	Yes	[52,54]
	**TERRITORIAL AND HIERARCHAL BEHAVIOURS**						
	Scent marking by chinning objects	Positive	Yes	No	No	Yes	[52]
	Cage guarding	Positive, Neutral, Negative	Yes	No	No	Yes	[52]
	Marking territory with urine or faeces (spraying) May be due to frustrated sexual behaviours of entire rabbits	Positive, Neutral, Negative	Yes	No	No	Yes	[52]
	**SOCIAL AND EXPLORATORY BEHAVIOURS**						
	Foraging	Positive	Yes	No	No	Yes	[52,54]
	Investigative behaviour	Positive	Yes	No	No	Yes	[52,54]
	Rearing or peri-scoping	Positive	Yes	No	No	Yes	[52,54]
	Digging or burrowing	Positive	Yes	No	No	Yes	[52]
	“Tooth purring” or “teeth chattering” Different from tooth grinding (bruxism)	Positive	Yes	No	No	Yes	[52]
	**RESTING BEHAVIOURS**						
	Sprawling or stretching out	Positive, Neutral	Yes	No	No	Yes	[52]
	Laying down or “flopped” on their side	Positive, Neutral	Yes	No	No	Yes	[52]
**Abnormal behaviours**	**ABNORMAL REPETITIVE BEHAVIOURS AND STEREOTYPIC BEHAVIOURS**						
	Excessive scratching or rubbing	Negative	Yes	No	No	Yes	[45,52]
	Barbering Can be self-inflicted or inflicted by cage-mates	Negative	Yes	No	No	Yes	[58]
	**ALTERED FOOD AND WATER CONSUMPTION**						
	Decreased food and water consumption Signs include refusal of usual fresh food or treats (for more than a day) No spillage of food or water (indicating consumption)	Negative, Neutral	Yes	No	No	Yes	[40,44,45,47,52]
	Prolonged duration of uneaten caecotrophs or inability to eat caecotrophs	Negative	Yes	No	No	Yes	[52]
	**ALTERED INTERACTIONS WITH HUMAN HANDLERS AND COMPANIONS** [41]						
	Hiding or refusal to leave hutch/hiding spots, or running away on approach	Negative	Yes	No	No	Yes	[40,50,52]
	Self-isolation	Negative	Yes	No	No	Yes	[46,50]
	Increased aggression to handler or companion	Negative	Yes	No	No	Yes	[40,50,52]
	**AGGRESSIVE BEHAVIOURS**						
	Grunting	Negative	Yes	No	No	Yes	[52]
	Thumping back legs	Negative	Yes	No	No	Yes	[40,52]
	Lunging	Negative	Yes	No	No	Yes	[52]
**Pain-related behaviours**	**BEHAVIOURS ASSOCIATED WITH PAIN**						
	Reduced or lack of responsiveness (including facing back of cage and immobility)	Negative	Yes	No	No	Yes	[51,52]
	Reluctance to move	Negative	Yes	No	No	Yes	[40]
	Loud tooth grinding (bruxism) Different from “tooth purring” or “teeth chattering”	Negative	Yes	No	No	Yes	[40,51,52]
	Failure to groom or over-grooming	Negative	Yes	No	No	Yes	[40,52]
	Squealing or shrieking	Negative	Yes	No	No	Yes	[42,52]
**(B) Guinea Pig animal-based welfare indicators grouped into physiological, behavioural and physical health and practical considerations.**							
**Body-system, Body part,** **Organ, or Descriptor of Indicator**	Animal-Based Welfare Indicators	Affective State	Easy To Train	High Costs (>AUD100)	Special Equipment	Time <5 min	Publications
**CATEGORY 1: PHYSIOLOGICAL**							
**Cardiovascular system**	Persistent increase or decrease or abnormality in heart rate	Negative	Yes	Yes—stethoscope	Yes—stethoscope	Yes	[59]
**Respiratory system**	Persistent decrease or increase in respiratory rate and effort	Negative	Yes	Yes—stethoscope	Yes—stethoscope	Yes	[60]
	Persistent presence of respiratory sounds (including coughing, sneezing, sniffling, wheezes, and crackles)	Negative	Yes	Yes—stethoscope	Yes—stethoscope	Yes	[60]
**Alimentary, gastrointestinal, digestive system**	Presence of diarrhoea or soft faeces with an unpleasant smell	Negative	Yes	No	No	Yes	[59,60]
	Changes in faecal output and appearance (including colour, consistency, frequency)	Negative	Yes	No	Yes—measuring utensils	Yes	[59,60]
**Urogenital system**	Poor breeding success (e.g., abortions, infertility)	Negative	No	Yes—ultrasound, radiographs, haematology, biochemistry, etc.	Yes—ultrasound, radiographs, haemotology, biochemistry, etc.	Yes	[60]
	Changes in urine output and appearance (incl. colour, consistency, frequency)	Negative	Yes	No	Yes—measuring utensils	Yes	[60]
**Musculoskeletal system**	Abnormal gait Includes weight shifting, stiff gait, shuffling gait, limping or lameness On palpation, joints may be hot and swollen	Negative	Yes	No	No	Yes	[59,60,61]
**Nervous system, adrenal glands**	Persistent increase or decrease in glucocorticoid levels and metabolites in comparison to normal	Negative, Neutral	No	Yes—corticosterone radioimmunoassay, etc.	Yes—corticosterone radioimmunoassay, etc.	No	[62]
**Body temperature**	Extreme changes in rectal temperature	Negative	Yes	No	Yes—thermometer	Yes	[60,63]
	Hot or swollen joints	Negative	Yes	No	No	Yes	[60]
**CATEGORY 2: PHYSICAL APPEARANCE**							
**Body weight**	Excess weight gain or loss	Negative	Yes	No	Yes—scales	Yes	[59,60,62]
**Body condition**	Body condition score	Negative, Neutral	Yes	No	No	Yes	[59,60,62]
**Integument system**	Unkempt, dirty, matted, erect coat or urine/faecal stained fur	Negative	Yes	No	No	Yes	[59,60]
	Piloerection	Negative	Yes	No	No	Yes	[64]
	Damage to fur or skin Includes active bleeding from lesions, ulcerations, old scabbed over/scaly lesions, cheilitis (ulcerative scabbing lesions around lips)	Negative	Yes	No	No	Yes	[59,60]
	Lumps in or under the skin	Negative	Yes	No	No	Yes	[59,60]
**Eyes, nose, ears, urogenital, mammary glands**	Presence of discharge from the eyes, nose, ears, mammary glands, urinary or genital organs	Negative	Yes	No	No	Yes	[59,60]
**Oral cavity and dentition**	Dental malocclusion, overgrowth, malformation, discolouration and loose, fracture or absent teeth	Negative	No	Yes—otoscope or oral speculum	Yes—otoscope or oral speculum	Yes	[59]
	Drooling (ptyalism) or wet chin	Negative	Yes	No	No	Yes	[59,60]
**Pain-related signs**	Closed eyes or squinting	Negative	Yes	No	No	Yes	[64]
**CATEGORY 3: BEHAVIOUR**							
**Natural behaviours**	Social activity	Positive	Yes	No	No	Yes	[65,66]
	Active for most of the 24 h period	Positive	Yes	No	No	Yes	[66]
	Scent marking	Positive	Yes	No	No	Yes	[66]
	Coprophagy	Positive	Yes	No	No	Yes	[61,65,66]
	Thigmotaxic behaviour	Positive	Yes	No	No	Yes	[66]
	Climb and jump	Positive	Yes	No	No	Yes	[61,66]
	Grooming	Positive	Yes	No	No	Yes	[65]
	Vocalisations	Positive, Negative, Neutral	Yes	No	No	Yes	[61]
	Eating and drinking	Positive	Yes	No	No	Yes	[61]
	Yawning	Positive	Yes	No	No	Yes	[61]
	**EXPLORATORY BEHAVIOURS**						
	Appropriate digging, walking, running, stretching, lying down, rearing, scratching, shaking, and standing	Positive	Yes	No	No	Yes	[61]
**Abnormal behaviours**	**ABNORMAL REPETITIVE BEHAVIOURS AND STEREOTYPIC BEHAVIOURS**						
	Excessive scratching or rubbing	Negative	Yes	No	No	Yes	[60]
	Barbering Can be self-inflicted or inflicted by cage-mates	Negative	Yes	No	No	Yes	[59]
	**ALTERED FOOD AND WATER CONSUMPTION**						
	Reduced or absence of food and water intake	Negative	Yes	No	No	Yes	[59,60,61]
**Pain-related behaviours**	**BEHAVIOURS ASSOCIATED WITH PAIN**						
	Decreased movement or reluctant to move Including forward or backward motion, turning the body or head, head or neck extension (rearing), ambulation	Neutral, Negative	Yes	No	No	Yes	[59,64,65]
	Decreased coprophagy	Neutral, Negative	Yes	No	No	Yes	[61,64,65]
	Biting, chewing or licking at enclosure	Neutral, Negative	Yes	No	No	Yes	[65]
	Changes in abdominal movements Including pressing abdomen to the floor (belly pressing), abdominal contraction with back arching, writhe (slow contortion of abdominal flank muscles), twitching (rapid muscle contraction of back muscles) and weight shifting.	Neutral, Negative	Yes	No	No	Yes	[61,64,65]
	Incomplete or abnormal behaviours or frequent abrupt ceasing movement	Neutral, Negative	Yes	No	No	Yes	[64]
**(C) Mice and rat animal-based welfare indicators grouped into physiological, behavioural, and physical health and practical considerations. Dark grey boxes represent welfare indicators displayed by rats; light grey represent welfare indicators displayed by mice; white boxes represent welfare indicators displayed by both rats and mice.**							
**Body-system, Body part, Organ, or Descriptor of Indicator**	Animal-Based Welfare Indicators	Affective State	Easy to Train	High Costs (>AUD 100)	Special Equipment	Time <5 min	Publications
**CATEGORY 1: PHYSIOLOGICAL**							
**Cardiovascular system**	Persistent increase or decrease or abnormality in heart rate	Negative	Yes	Yes—stethoscope	Yes—stethoscope	Yes	[67,68,69,70,71]
	Presence of heart murmur or arrhythmia	Negative, Neutral	Yes	Yes—stethoscope	Yes—stethoscope	Yes	[67,68,69,70,71]
**Respiratory system**	Persistent decrease or increase in respiratory rate and effort (laboured breathing/respiratory distress/dyspnoea/apnoea/abdominal breathing)	Negative	Yes	Yes—stethoscope	Yes—stethoscope	Yes	[67,68,69,70,71,72,73]
	Persistent presence of respiratory sounds (including coughing, sneezing, sniffling, wheezes, and crackles)	Negative	Yes	Yes—stethoscope	Yes—stethoscope	Yes	[67,68,70,71]
**Alimentary, gastrointestinal, digestive system**	Presence of diarrhoea or soft faeces with an unpleasant smell	Negative	Yes	No	No	Yes	[67,68,70,71]
	Changes in faecal output and appearance (including colour, consistency, frequency)	Negative	Yes	No	Yes—measuring utensils	Yes	[67,68,69,72]
**Urogenital system**	Changes in urine output and appearance (including colour, consistency, frequency)	Negative	Yes	No	Yes—measuring utensils	Yes	[67,68,69,72]
**Musculoskeletal, nervous and vestibular system**	Abnormalities in gait Gait score Abnormalities include lack of balance, stumbling, stiff-legged gait or reluctance to move Lameness	Negative	Yes (variable)	No	No	Yes	[68,69,70,71,72,74,75,76,77]
**Nervous system, adrenal glands**	Persistent increase or decrease in glucocorticoid levels and their metabolites in comparison to normal.	Negative, Neutral	No	Yes—corticosterone radioimmunoassay, etc.	Yes—corticosterone radioimmunoassay, etc.	No	[67,68,69,72,78,79]
**Body temperature**	Extreme changes in body temperature	Negative	Yes	No	Yes—thermometer	Yes	[67,68,72]
**Hydration status**	Dehydration	Neutral, Negative	Yes	No	No	Yes	[67,68,72,74]
**CATEGORY 2: PHYSICAL APPEARANCE**							
**Body weight**	Excess weight gain or loss	Negative	Yes	No	Yes—scales	Yes	[68,69,70,71,72,74,77,80,81,82,83]
**Body condition**	Body condition score	Negative, Neutral	Yes	No	No	Yes	[68,69,70,71,72,74,77,80,81,82,83]
**Integument system**	Unkempt, dirty, matted, erect coat or urine/faecal stained fur	Negative	Yes	No	No	Yes	[68,69,70,71,72,77,84]
	Piloerection	Negative, Neutral	Yes	No	No	Yes	[68,70,73,74,77,84]
	Damage to fur or skin Including the presence of bite wounds, discolouration, lesions such as sores, scabs, scales, ulcerations, crusting and areas of hair loss	Negative	Yes	No	No	Yes	[67,68,69,70,71,72,73,74,77,84]
	Lumps under the chin or mandible	Negative	Yes	No	No	Yes	[70,71,72]
	Lumps in or under the skin	Negative	Yes	No	No	Yes	[70,71,72]
	Excessively loose skin	Negative	Yes	No	No	Yes	[70,71,72]
**Eyes, nose, urogenital, mammary glands**	Presence of discharges from the eyes, nose, mammary glands, urinary or genital organs	Negative	Yes	No	No	Yes	[69,70,71,72,74]
**Posture**	Altered abnormal postures Hunched back, arched back with front paws tucked under the body, a lowered head and a tucked abdomen Prostration or extension	Negative	Yes	No	No	Yes	[68,69,70,71,72,74,77,84]
**Abdomen**	Swollen or distended abdomen	Negative	Yes	No	No	Yes	[69,72,74]
**Eyes and lacrimal glands**	Partially closed, sunken, or dull eyes	Negative	Yes	No	No	Yes	[68]
	Chromodacryorrhea (porphyrin staining)	Negative	Yes	No	No	Yes	[68,69,77,85]
**Pain-related signs**	Facial grimacing (Grimace scale) **Grimace scale for Rats—Indicators:** Orbital tightening Nose/cheek flattening Ear changes Whisker change **Grimace scale for Mice—Indicators:** Orbital tightening Nose bulge Cheek bulge Ear position Whisker change	Negative	Yes	No	No	Yes	[32,33,53,67,68,69,72,74,77,86,87,88,89,90,91,92,93,94,95]
	Increased composite pain score	Negative	Yes	No	No	Yes	[68,88,95,96,97,98,99]
**CATEGORY 3: BEHAVIOURIAL**							
**Natural** **behaviours**	Nocturnal/crepuscular behaviour	Positive	Yes	No	No	Yes	[100,101,102]
	Avoidance of open spaces	Positive	Yes	No	No	Yes	[100,101,102]
	**EXPLORATORY BEHAVIOURS**						
	Appropriate running, jumping, climbing, sniffing, stretching, foraging, digging, rearing, resting, coprophagy, and chewing,	Positive, Neutral	Yes	No	No	Yes	[67,68,73,74,77,86,103]
	Burrowing	Positive, Neutral	Yes	No	No	Yes	[104,105,106]
	**SOCIAL AND PLAY BEHAVIOURS** [49,74]						
	Object manipulation	Positive, Neutral	Yes	No	No	Yes	[68,107]
	Rough-and-tumble play with cage mates and human handlers	Positive, Neutral	Yes	No	No	Yes	[68,107]
	Social activity	Positive	Yes	No	No	Yes	[77,100,101,102]
	Positive behaviour	Positive	Yes	No	No	Yes	[67,74,108]
	**GROOMING**						
	Self-grooming and allo-grooming with cage mates	Positive	Yes	No	No	Yes	[68,73,77,86]
**Abnormal** **behaviours**	Overgrooming	Negative	Yes	No	No	Yes	[68,70,73,84,86,109,110,111]
	**ALTERED FOOD AND WATER CONSUMPTION**						
	Reduced or absence of food and water intake	Negative	Yes	No	No	Yes	[68,72,73,80,82,83,84,112]
	**ABNORMAL REPETITIVE AND STEREOTYPIC BEHAVIOURS**						
	Locomotor or oral repetitive behaviours	Negative	Yes	No	No	Yes	[68,113]
	Stereotypic behaviour which may be sex and strain related	Negative	Yes	No	No	Yes	[67,73,77,114,115,116]
	Barbering Self-inflicted or inflicted by cage-mates.	Negative	Yes	No	No	Yes	[67,69,72,73,77,117]
	**ALTERED SOCIAL BEHAVIOURS**						
	Self-isolation	Negative	Yes	No	No	Yes	[68,84,118,119]
	Frequent agonistic severe behaviour towards cage mates	Negative	Yes	No	No	Yes	[68,73,74,77,84,118,119]
	**ALTERED ACTIVITY LEVELS**						
	Altered, increased or decreased activity	Negative	Yes	No	No	Yes	[68,73,74,77,82,84,104,105,112]
	Altered, increased or decreased alertness	Negative, Neutral, Positive	Yes	No	No	Yes	[69,72]
	Voluntary frequent wheel running	Negative	Yes	No	No	Yes	[120]
	**ALTERED INTERACTIONS WITH HUMAN HANDLERS**						
	Avoidance or agonistic behaviour towards human handlers Latency to approach	Negative	Yes	No	No	Yes	[68,69,74]
	Urinating or defecating during handling	Negative	Yes	No	No	Yes	[69,72,74]
	**ALTERATIONS IN SOUND WAVE FREQUENCY OF VOCALISATIONS** [40,49,50,53,112]						
	20 kHz vocalisations	Negative, Neutral	Variable	Yes	Yes—sound level meter	Yes	[68,72,73,74,86,121,122]
	50 kHz vocalisations	Positive, Neutral	Variable	Yes	Yes—sound level meter	Yes	[68,72,73,86,121,122,123]
	**ALTERATIONS IN BURROWING BEHAVIOUR**						
	Decreased or absence spontaneous burrowing behaviour	Negative, Neutral	Yes	No	No	Yes	[67,68,86,103,104,105,106,120,124,125,126]
	**BREEDING-RELATED BEHAVIOURS**						
	Excessive cornering, or wall-hugging behaviour	Negative, Neutral	Yes	No	No	Yes	[68,69,77,127]
	Nest building performance Time to integrate into nest and nesting consolidation scoring	Positive, Neutral, Negative	Yes (variable)	No	No	Yes	[67,69,73,74,86,106,128,129,130]
	Nursing, pup retrieval, and interaction with pups after nest disturbance	Positive, Neutral	Yes	No	No	Yes	[19]
	Pup–pup interactions						
	Pup–adult interactions						

Dark grey boxes represent welfare indicators studied to be displayed by rats; light grey represent welfare indicators studied to be displayed by mice; white boxes represent welfare indicators studied to be displayed by both rats and mice.

**Table 3:** Summarises the number of welfare indicators for each species as per one of three categories of animal-based welfare indicators.

	Animal			
Category of Animal-Based Welfare Indicators	Rabbits	Guinea Pigs	Rats and Mice	Total Number of Indicators for Each Category
Physiological indicators	17	11	11	39
Physical health indicators	27	10	15	52
Behavioural indicators	31	28	40	99
**Total number of indicators for each animal**	75	49	66	190

**Table 4:** Shared welfare indicators across guinea pigs, mice, rabbits, and rats categorised in physiological, physical health and behavioural.

Category of Animal-Based Welfare Indicators	Similar Animal-Based Welfare Indicators Shared across Rabbits, Guinea Pigs, Rats and Mice
Physiological indicators	Persistent changes (especially increases) in heart rate Persistent changes (especially increases) in respiratory rate and effort Persistent presence of respiratory sounds Presence of diarrhoea or prolonged production of soft stools with unpleasant smell Changes in faecal output and appearance (colour, consistency and frequency) Changes in urine output and appearance (colour, consistency and frequency) Abnormal gait Persistent changes (especially increases) in glucocorticoid levels and their metabolites (e.g., corticosterone and cortisol) Extreme changes in body temperature
Physical health indicators	Body weight changes Body condition score Unkempt, dirty, matted, erect coat or urine/faecal stained fur Piloerection Damage to fur or presence of skin lesions Lumps in or under the skin Presence of discharge from the eyes, nose, ears, mammary glands, urinary or genital organs Altered posture
Behavioural indicators	Social behaviours Play behaviours Exploratory behaviours Grooming (allo-grooming, self-grooming, mutual grooming) Coprophagy Vocalisations Avoidance of open spaces (thigmotaxic behaviour) Abnormal repetitive behaviours and stereotypic behaviours—e.g., barbering Altered food and water consumption Pain-associated behaviours—e.g., reluctance to move, reduced responsiveness, over-grooming, or failure to groom

**Table 5:** Simple comparison of binary and numerical systems for animal welfare indicators with advantages and disadvantages.

	**Advantages**	**Disadvantages**
**Binary**	Less time consuming Easier to use as based Potential for less subjective assessment May be more consistent if descriptions are clear	Limited description of intensity or severity of the indicator Potential less complexity of data
**Numerical**	Severity and intensity can be scored Potential for detailed data to be collected Allows for tracking of trends over time	Potential for more subjectivity when assigning scores Can be time consuming Scores can under- or overestimate the severity of an indicator

## Data Availability

Not applicable.
